# Environmental and social correlates of the plumage color polymorphism in an urban dweller, feral pigeon (*Columba livia* f. *domestica*)

**DOI:** 10.1038/s41598-024-82937-z

**Published:** 2024-12-28

**Authors:** Piotr Skórka, Beata Grzywacz, Michał Bełcik, Piotr Tryjanowski

**Affiliations:** 1 Institute of Nature Conservation, Polish Academy of Sciences, Adama Mickiewicza 33, 31-120 Kraków, Poland; 2 Institute of Systematics and Evolution of Animals, Polish Academy of Sciences, Sławkowska 17, 31-016 Kraków, Poland; 3 Institute for Advanced Study, Technical University of Munich, 85748 Garching, Germany; 4 Department of Zoology, Poznań University of Life Sciences, Wojska Polskiego 71C, 60-625 Poznań, Poland

**Keywords:** Birds, Color polymorphism, Plumage diversity, Urbanization, Urban ecology, Social evolution, Genetic variation, Behavioural ecology

## Abstract

We examined how urban environments affect the abundance, proportion, and diversity of plumage color morphs in feral pigeons. Five major plumage color morphs (black, blue, white, red, and mixed) were counted in sixty 25-ha plots in Poznań City (Poland). Generalized additive models were used to study the correlations among abundance, proportion of morphs, and environmental factors. Anthropogenic food sources were positively correlated with the abundance of black morphs and the proportions of black and red morphs. The blue morph abundance peaked at a moderate percentage of tall building cover, but its proportion decreased. A similar decrease was observed in the mixed plumage morphs. The abundance of blue morphs decreased, whereas the abundance of white morphs and the proportion of red morphs increased as the distance from the city center increased. The plumage color morph diversity (Simpson) index was positively correlated with food sources and hedgerow density but negatively correlated with street density. Color morph diversity in the study area may be sustained by differential responses of morphs to the environmental features of the urban environment. However, the positive correlation between the abundance of morphs indicates social attraction rather than social isolation among plumage color morphs.

## Introduction

Birds exhibit notable variations in plumage patterns, which are essential in their evolution, ecology, and behavior^1,2^. The size, shape, location, and regularity of the plumage patches act as signals for individual birds of the same and other species^3^. Plumage color patterns provide information about an individual’s age, sex, identity, social status, breeding conditions, and other characteristics mediating bird interactions^1,4,5^.

In addition to social factors, biogeographic factors notably influence plumage variation^6^. Birds are lighter in warmer areas following Bogert’s rule and darker in wetter locations; however, they also exhibit lighter coloration at extremely low temperatures^7^. Moreover, a positive correlation exists between range size and color variability in parrots and passerines^8^. This finding supports the theory that plumage variation and adaptation of bird species are connected^9^. However, local environmental influences, particularly urbanization, frequently alter the biogeographic patterns of color variation in birds^10^.

Urbanization is accelerating worldwide, transforming landscapes, influencing phenotypes, and affecting bird fitness^11,12^. Urbanization also influences plumage coloration, promoting the prevalence of species with gray coloration^13^. Furthermore, bird species categorized as “urban exploiters” typically have uniform plumage, whereas those categorized as “urban avoiders” exhibit diverse plumage^14^. This can also be confirmed by intraspecific color plumage variation. Great tits (*Parus major*) are paler in urban environments than in forest habitats, a phenomenon called “urban dullness”^15^. Surmacki et al.^16^ demonstrated that the plumage color intensity decreases in urban areas near roads with high traffic volumes and increases in rural areas. According to a long-term study by Smith et al.^17^, white-crowned sparrows (*Zonotrichia leucophrys*) in urban areas experience a gradual change in their dorsal plumage, becoming darker and duller. In contrast, sparrows from rural populations in nearby areas maintain plumages with more intense hues and greater complexity^17^.

**Fig. 1:**
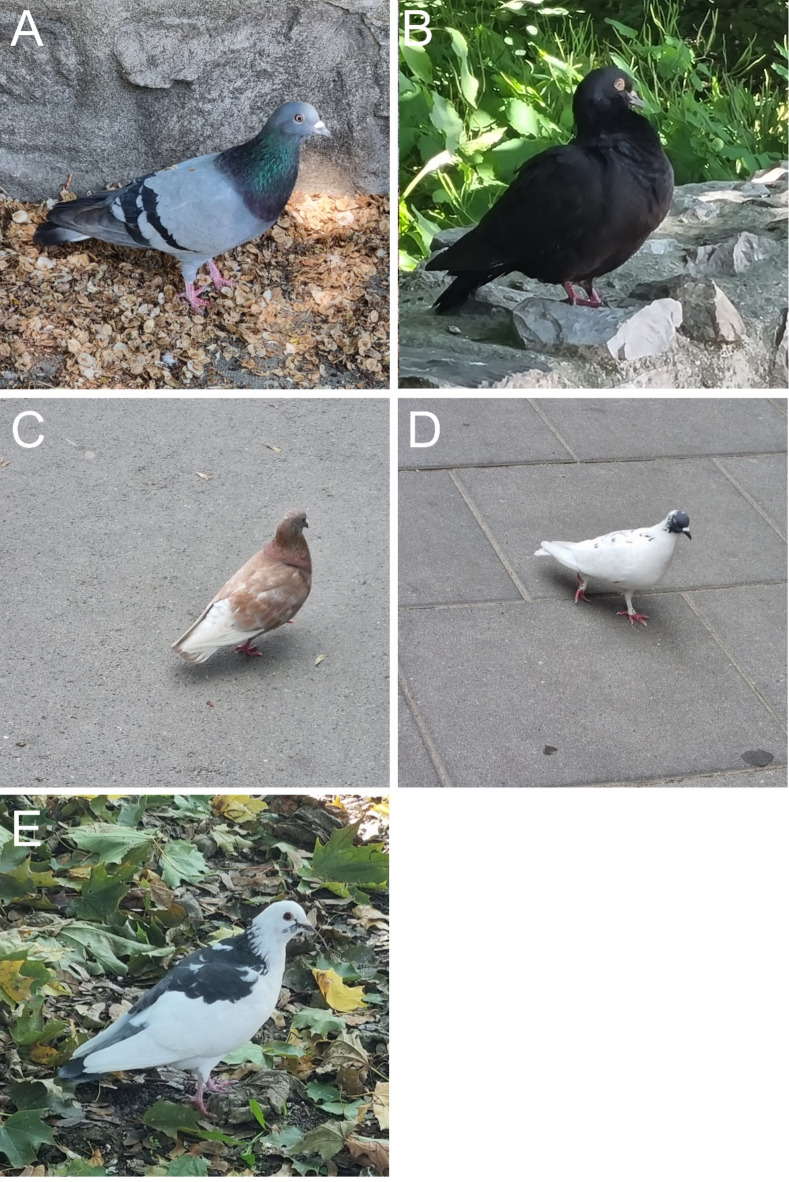
Examples of plumage color morphs in feral pigeons: (**A**) blue, (**B**) black, (**C**) red, (**D**) white, and (**E**) mixed. Photos by Piotr Skórka.

The feral pigeon *Columba livia* f. *domestica*, a successful urban exploiter, exhibits a wide range of plumage colors (Fig. 1) primarily because of two types of melanin pigments: eumelanin, which produces black and gray colors, and pheomelanin, which is responsible for (ash) red hues^18–20^. Eumelanin and pheomelanin plumage colors in feral pigeons are highly heritable (heritability of melanin degree: 0.82 ± 0.12^21^). Changes in the melanocortin-1 receptor gene (MC1R), responsible for plumage variation in other bird species, are not the only source of diverse plumage hues in feral pigeons. Several loci control pigeon plumage color gene expression, suggesting polygenic inheritance^21,22^. The Checker and T-pattern alleles, which affect plumage patterning, exhibit a dominance hierarchy^23^. White or albino phenotypes can result from MC1R gene mutations that deplete eumelanin^24^. Furthermore, introgression from domestic breeds contributes to the diversity of plumage patterns observed in feral pigeons because selective breeding practices have introduced new alleles into the gene pool^21,25–28^.

Environmental factors also notably influence the expression of plumage colors. The urban environment may be particularly favorable for certain plumage color morphs^29,30^. Additionally, feather health, which can be affected by bacterial load and exposure to pollutants, may influence the appearance of plumage^31,32^. Black pigeons exhibit lower susceptibility to parasites, which may further affect their survival and reproductive success in urban environments^33^. Therefore, studying plumage variation within this species is valuable for investigating how the urban environment affects color polymorphism in populations^1,34^.

In this study, we investigated whether the abundance and proportion of different colored plumage morphs in feral pigeons are similarly affected by the urban environment or whether urbanization differentially affects the abundance and proportion of each colored morph. We used abundance in a plot and proportion (relative abundance) because both are interrelated and fully describe population functioning^35,36^. Although models for these estimates should produce similar results, abundance may better describe associations with environmental variables, while proportion may more effectively show interactions among plumage morphs, as observed in multi-species communities^37,38^. Urbanization is a multifaceted phenomenon, with many variables potentially affecting species populations. Thus, making specific predictions about how different plumage color morphs respond to environmental factors associated with urbanization is difficult. Several factors (anthropogenic and natural food sources and the availability of nesting sites) should positively correlate with the abundance and proportion of all morphs. In contrast, black (melanistic) morphs in birds can be more aggressive or active^39,40^ and thus have higher energy expenditure and greater food consumption and energy needs than non-melanistic individuals^40^. Therefore, a positive correlation between the food source and black morph abundance is anticipated. Traffic volume is high in urban environments and may be a major factor associated with road mortality in birds^41,42^. Thus, one may predict that street density will have a negative effect on the blue and black morphs primarily due to their increased likelihood of collisions with vehicles, as their coloration closely resembles that of asphalt. Red or white morphs may be visible to car drivers, who can easily adjust their speed to avoid collisions. However, this prediction may be complicated by the behavioral response of birds, which may avoid traffic noise, leading to a decrease in the overall abundance of all morphs on roads with high traffic volumes, as has been reported for many different species^43,44^.

Generally, as feral pigeons are urban dwellers, the abundance and proportion of all plumage color morphs should be the highest in the city center. Feral pigeons are typically sedentary in urban environments and cover small distances. For example, in Basel, Rose et al.^45^ found that 32% of the pigeons remained within 0.3 km of the home lofts, and only 7.5% traveled distances greater than 2 km. This may have led to a high spatial autocorrelation in the abundance and proportion of all plumage color morphs. Moreover, social factors may influence the abundance and proportion of plumage color morphs. Mating in feral pigeons tends to be highly disassortative^46^, indicating that individuals may prefer different color morphs. Consequently, a positive correlation between the abundance of color morphs was expected. Finally, environmental and social variables that increase the proportion of the most abundant plumage color morph and decrease the least abundant should negatively affect the plumage color morph diversity index, whereas variables that are positively associated with the abundance of the less abundant morphs should have a positive effect on the color plumage diversity index because the diversity index is the highest when the abundances (or proportions) of all plumage morphs are similar.

## Methods

### Study area

The study was conducted in 2010 in Poznań (western Poland), a prominent Polish city with a population of 556,000 and an area of 261.3 km^2^. This translates to a population density of 2,123 persons /km^2^. The altitude varies between 60 and 157 m. The climate in Poznań is characterized as a humid continental climate, with mild winters and relatively hot summers. The average temperature in the coldest month, December, is 0.2 °C, whereas in the hottest month, June, it is 17.4 °C. The annual precipitation is approximately 500 mm^47^.

### Color morphs

Similar to earlier studies^21,28,48^ the following color morphs (categories) of feral pigeons were counted: black (melanic), blue (wild-type), white (or almost white), red (also called ash-red or brown), and mixed pigeons that could not be classified into the preceding categories (Fig. 1). The first three categories correspond to a decrease in eumelanin production, whereas the red category corresponds to pheomelanin production^21^. More plumage categories were within each morph based on the distribution of color patches; however, we counted these five major color morphs because our study included several volunteers. Preliminary observations indicated that further categorization of the plumage pattern, particularly in the blue morph, was challenging and often impractical because pigeons frequently rested on rooftops, lofts, and windowsills, making detailed plumage pattern classification difficult.

### Estimation of pigeons’ abundance

We selected 60 plots of 0.5 × 0.5 km (25 hectares) in the city’s residential sections (with houses and apartment blocks) to determine the number of feral pigeons^47^. Plots were randomly selected based on the geographical coordinates of the places that served as the upper-left corners of the square plots. The mean distance between the borders of the nearest plots was 1050 m (minimum: 122 m; maximum: 2745 m). Selection was performed using Quantum 1.5 GIS software. The study was conducted between early June and July. The specified time frame corresponded to the highest level of reproductive activity of this species in Poznań, as reported by Dabert^49^. Three counts were conducted in each plot with an interval of approximately 10 days between each count. All visits lasted for an hour and started between 6 a.m. and 6 p.m. Consecutive visits to the plots began at different times. Observations were conducted under good weather conditions characterized by the absence of rain and strong winds. Twenty trained observers participated in the field surveys. Each observer conducted three surveys, which were always in the same plot. On an average (median), each observer surveyed three plots (1–5 plots). The observers conducted a comprehensive visual survey of the entire study area by walking through individual plots. The number of pigeons of each morph observed inside the plot area was counted, excluding any flocks that passed through the airspace above. Binoculars were used to survey all buildings and locate pigeons on rooftops or windowsills. We also explored all locations where birds congregated to find food. The observers refrained from counting pigeons that were suspected of having already been counted (based on the movement of birds in relation to the observer’s path and plumage). Each observer was provided with printed maps of the plot, where survey details were noted along with certain environmental variables, such as hedgerows, schools, food resources, and tall buildings. This was performed to improve the measurement of environmental variables from aerial photographs in GIS.

### Environmental explanatory variables

The explanatory environmental variables that could affect pigeon abundance^43^ were measured in each plot.

1. The quantities of anthropogenic food sources (Food sources in Table 1). We counted the number of grocery stores and fast food restaurants, the number of litter bins (of any type), and the locations of all bird-feeding spots (based on first-hand observations of people feeding on birds and leaving food remnants). These components were summed to obtain the total number of food sources available to pigeons.
2. Street density (m/10 ha, Street density in Tables 1 and 2). Traffic may influence pigeon mortality through collisions with vehicles^50^.
3. Hedgerow density (m/10 ha, Hedgerows in Tables 1 and 2). During preliminary observations, pigeons often used hedgerows to find food. Therefore, we hypothesized that there would be a positive correlation between the abundance of hedgerows and pigeons.
4. Percentage of plots covered with green areas (Green areas in Tables 1 and 2). All parks, squares, lawns, and barren areas found in residential neighborhoods were labeled as green spaces.
5. Number of schools (primary, secondary, and high) in each plot (Schools in Tables 1 and 2). In Polish schools, students customarily hang bird feeders and feed birds. Schools frequently have substantial green spaces that serve as feeding and resting sites for pigeons.
6. Percentage of plots covered by tall buildings of more than four stories (Tall buildings in Tables 1 and 2). We anticipated a positive correlation between this variable and the plumage morph abundance. This is because pigeons are more likely to be found in locations with windowsills or where food is tossed from the windows. We also recorded the percentage cover of low-rise buildings (family residences); however, we did not use this variable because it was strongly correlated with the cover of tall buildings^47^.
7. Distance (km) from the city center, defined as the central square in the Old City district (Dist. city center; in Tables 1 and 2).
8. Geographical coordinates (Longitude and latitude in Tables 1 and 2).

**Table 1:** Effect of environmental variables on feral pigeon plumage color morphs’ abundance and diversity (Simpson index). Results from generalized additive models with negative binomial and beta (for diversity) error distributions. Explanations:* SE* standard errors, *edf* effective degrees of freedom, *P* statistical significance. *Edf* equal or lower than one indicates a linear relationship, and above one indicates a non-linear relationship. Statistically significant effects have *Edf* and *P* emboldened. Significant* P* means that the probability of receiving the result by chance is lower than 0.05.﻿

Parametric terms	Blue	*P*	Black	*P*	Red	*P*	White	*P*	Mixed	*P*	Diversity	*P*
**Intercept (SE)**	**2.723** **(0.117)**	**< 0.001**	**0.883 (0.136)**	**< 0.001**	0.096 (0.153)	0.630	-0.144 (0.161)	0.370	0.204 (0.165)	0.216	**0.605 (0.008)**	**< 0.001**
**Smooth terms**	*Edf*	*P*	*Edf*	*P*	*Edf*	*P*	*Edf*	*P*	*Edf*	*P*	*Edf*	*P*
Food sources	0.000	0.637	**1.453**	**0.003**	0.000	0.804	0.369	0.235	0.000	0.386	**2.894**	**< 0.001**
Density of streets	0.893	0.099	0.000	0.525	0.000	0.626	0.199	0.282	0.994	0.094	**2.754**	**< 0.001**
Hedgerows	0.000	0.818	0.000	0.631	0.000	0.498	0.000	0.876	0.000	0.690	**1.280**	**< 0.001**
Green areas (%)	0.000	0.636	0.000	0.356	0.000	0.770	0.000	0.937	0.438	0.250	**1.633**	**0.049**
Schools	0.373	0.181	0.000	0.519	1.176	0.106	0.000	0.867	0.666	0.077	0.000	0.745
Tall buildings (%)	**1.352**	**0.047**	0.073	0.291	0.680	0.075	0.000	0.375	**0.827**	**0.016**	0.450	0.164
Dist. city center	**0.811**	**0.018**	0.000	0.966	0.000	0.563	**1.867**	**< 0.001**	0.000	0.942	0.567	0.198
Longitude*Latitude	0.000	0.873	**1.508**	**0.011**	**1.401**	**0.023**	**6.080**	**< 0.001**	**1.302**	**0.031**	0.000	0.381
Other morphs	**2.380**	**< 0.001**	**2.231**	**< 0.001**	**1.874**	**< 0.001**	**2.159**	**0.001**	**2.109**	**< 0.001**	–	–
Total abundance	–	–	–	–	–	–	–	–	–	–	**2.956**	**< 0.001**
**Deviance explained (%)**	52.6	–	73.3	–	56.2	–	71.6	–	63.3	–	99.4	–

**Table 2:** Effect of environmental variables on the proportion of feral pigeon plumage color morphs. Results from generalized additive models with beta error distributions. Explanations:* SE* standard errors, *edf * effective degrees of freedom, *P* statistical significance. *Edf* equal or lower than one indicates a linear relationship, and above one indicates a non-linear relationship. Statistically significant effects have *Edf* and *P* emboldened. Significant *P* means that the probability of receiving the result by chance is lower than 0.05.

Parametric terms	Blue	*P*	Black	*P*	Red	*P*	White	*P*	Mixed	*P*
Intercept (SE)	**1.328 (0.125)**	**< 0.001**	**-2.221 (0.123)**	**< 0.001**	**-2.522 (0.138)**	**< 0.001**	**-1.578 (0.156)**	**< 0.001**	**-2.901 (0.134)**	**< 0.001**
**Smooth terms**	*Edf*	*P*	*Edf*	*P*	*Edf*	*P*	*Edf*	*P*	*Edf*	*P*
Food sources	0.000	0.523	**1.662**	**< 0.001**	**1.900**	**< 0.001**	0.000	0.926	0.000	0.682
Density of streets	0.547	0.201	0.000	0.673	0.430	0.189	0.123	0.245	0.428	0.252
Hedgerows	0.000	0.943	0.000	0.663	0.000	0.686	0.291	0.262	0.814	0.148
Green areas (%)	0.704	0.065	0.000	0.759	0.000	0.655	0.000	0.524	0.000	0.867
Schools	0.308	0.207	0.000	0.737	1.307	0.117	0.000	0.619	0.000	0.512
Tall buildings (%)	**1.857**	**< 0.001**	0.000	0.345	1.148	0.103	0.000	0.788	0.000	0.621
Dist. city center	0.000	0.771	0.000	0.957	**2.534**	**< 0.001**	0.000	0.848	0.000	0.723
Longitude*Latitude	**1.766**	**< 0.001**	**1.685**	**0.001**	**3.751**	**< 0.001**	0.988	0.123	1.093	0.098
Total abundance	0.000	0.648	**1.544**	**0.008**	0.501	0.150	0.242	0.251	**1.727**	**< 0.001**
**Deviance explained (%)**	73.1	–	70.1	–	72.9	–	45.0	–	45.0	–

### Data handling, visualization, and statistical analysis

We used the number of pigeon plumage morphs recorded in each survey to estimate abundance. For the calculations, we used the mean abundances from three surveys. This mean abundance was rounded to facilitate the use of Poisson family distributions in the statistical analyses, which is more appropriate for count data than a Gaussian distribution.

The abundance and diversity of pigeon plumage color morphs were interpolated to the city area using the Kriging tool in ArcGIS^51^.

Statistical analyses were performed using the R software (ver. 4.3.0) programming language^52^. We correlated variables describing the characteristics of the urban environment with the mean abundance, proportion and diversity of pigeon plumage morphs. We used generalized additive models (GAMs) with negative binomial (abundance) or beta (proportion and morph diversity) error distribution implemented in the “mgcv” package^53^. We compared the Akaike information criterion (AIC) for abundance models with negative binomial distributions to AIC models with a Poisson error distribution; the latter provided a consistently worse fit (higher AIC values). The proportion of each morph was calculated as the mean abundance of plumage color morphs divided by the sum of the abundances of all morphs. We used the reciprocal Simpson index (a continuous variable ranging from 0 to 1), which is the probability that two randomly selected pigeons belong to different morphs, to calculate the plumage color morph diversity. Therefore, GAMs with beta error distribution were used.

The following independent explanatory environmental variables were used in all the GAMs: number of anthropogenic food sources (log-transformed), street density, hedgerow density, cover of green areas (%), number of schools, cover of tall buildings (%), and distance (km) from the city center. They were modeled as splines to enable non-linear associations. A natural logarithmic transformation of the number of anthropogenic food sources was used to minimize the effects of detached observations. The geographic coordinates (longitude and latitude) of the plot were fitted as the interaction of the regression splines in the GAMs to control for the spatial autocorrelation of the data; thus, part of the variation in the response variables was explained by the geographical location. Moreover, the abundance of other morphs was included as covariates in the GAMs built for each morph. In the GAMs explaining proportion of each morph and plumage diversity, the total abundance (the sum of the abundances of all morphs) was also included as a covariate. We applied variable selection (using gam function argument select equaling “TRUE”) to all GAMs. This imposed additional penalties on the null spaces for all smoothing processes in the current model. Adding a penalty to the null space of the smooths in the model allowed these functions to be penalized during fitting, which can result in shrinkage of specific terms. Marra and Wood^54^ concluded that an additional penalty term in the smoothness-selection procedure yielded the best results.

### Ethical statement

This research was conducted in accordance with the relevant regulations and Polish law. Surveys were conducted in publicly available spaces and did not involve bird capture or chasing. Informed consent was obtained from each observer to conduct the field surveys.

## Results

**Fig. 2:**
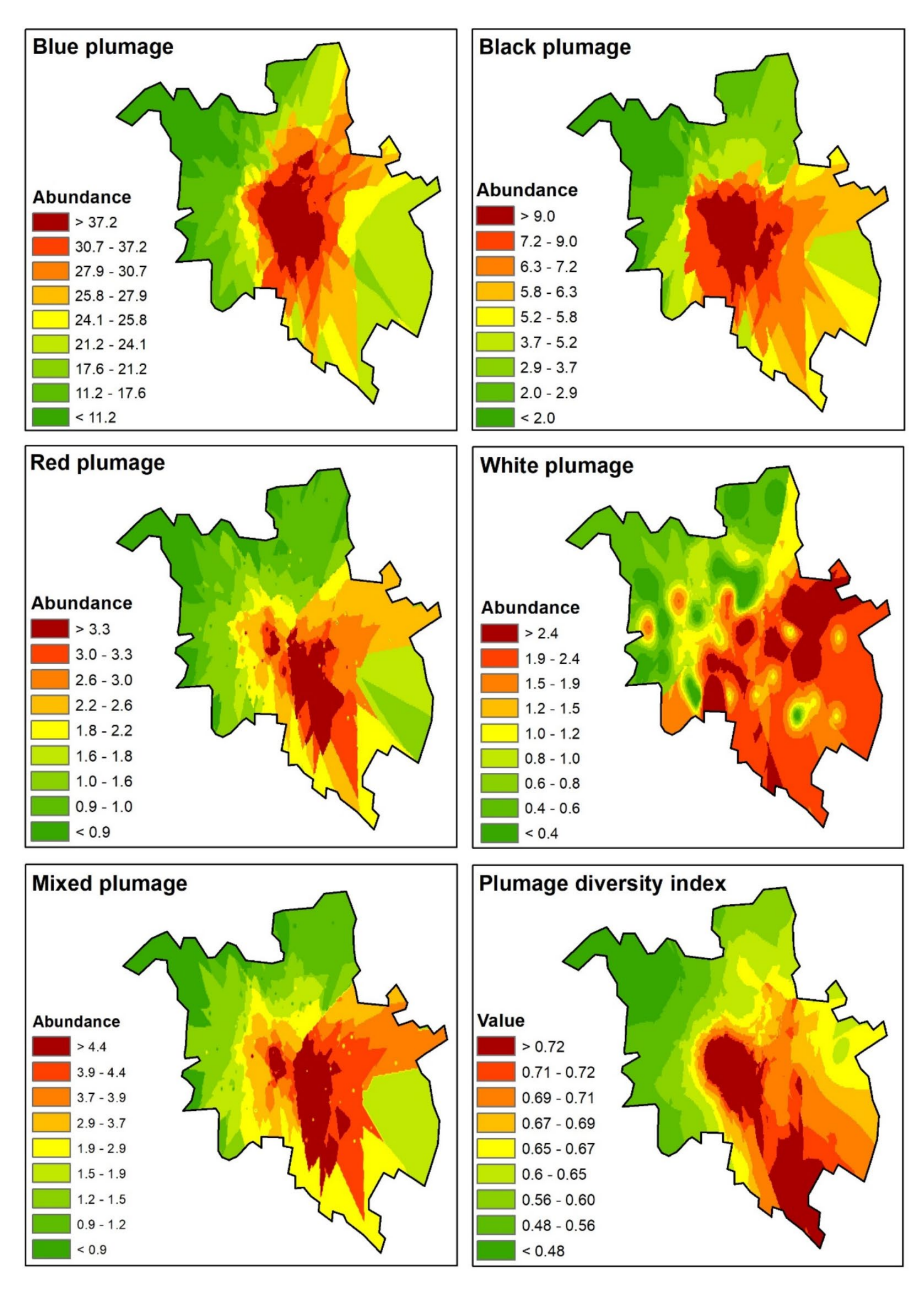
Interpolated abundance of different plumage color morphs of feral pigeon and their diversity index in Poznań. Calculations were done in ArcGIS Desktop: Release 10.8.2 (ESRI 2024). Available from https://www.arcgis.com/.

During the three surveys, 5959 pigeons were observed, comprising 4215 (70.7%) blue plumage morphs, 897 black plumage morphs (15.1%), 269 (4.5%) red plumage morphs, 208 (3.5%) white morphs, and 370 (6.2%) pigeons with mixed plumage coloration. The highest abundance was observed in the blue plumage morph (mean = 12.4 individuals/plot/survey; minimum = 0; maximum = 93), followed by the black (mean = 3.5; minimum = 0; maximum = 27), red (mean = 2.1; minimum = 0; maximum = 14), and white morphs (mean = 2.0; minimum = 0; maximum = 10). The mixed plumage morphs had an abundance of 2.3 individuals/plot (minimum = 0; maximum = 30). The abundance of morphs was concentrated around the city center (Fig. 2); however, spatial differences were observed in the distribution of plumage color morphs. For example, white plumage morphs mainly occurred in the southeastern part of the city.

### Variables correlated with the abundance of plumage color morphs

**Fig. 3:**
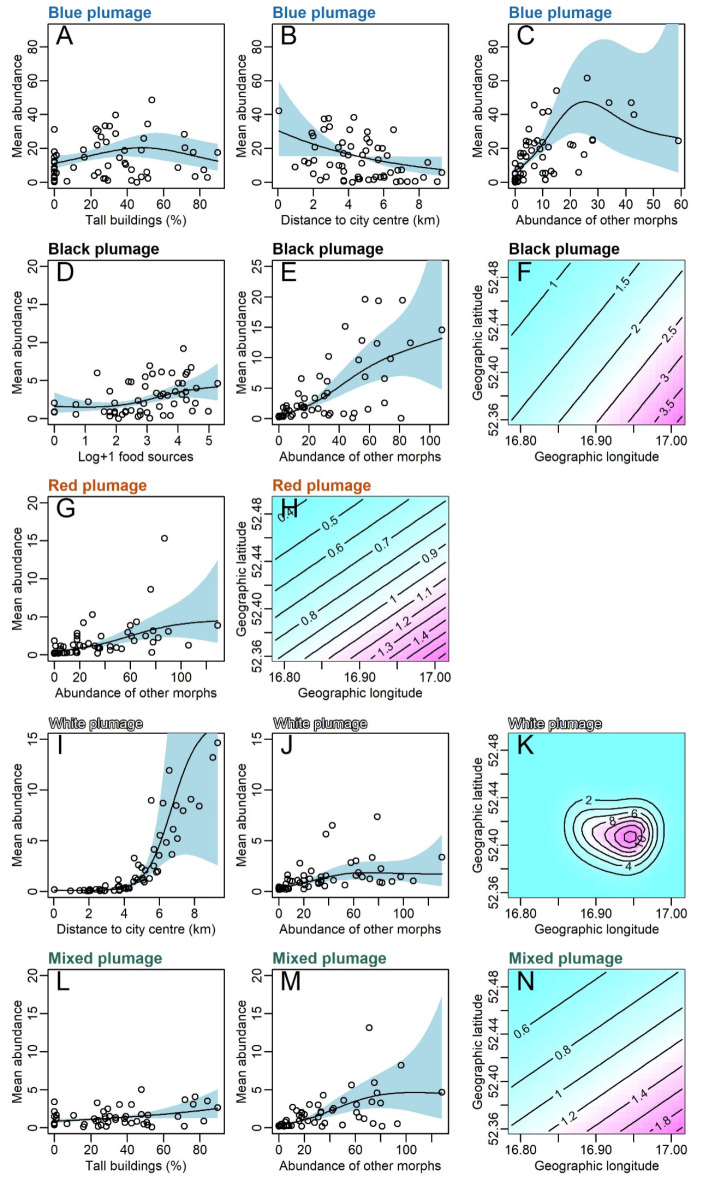
Variables correlated with the abundance of blue plumage morph (**A**–** C**), black plumage morph (**D**–** F**), red plumage morph (**G**,**H**), white plumage morph (**I**–**K**), and mixed plumage morph (**L**–**N**). Results from generalized additive models with negative binomial error distribution (Table 1). Isolines in graphs F, H, K, and N indicate changes in the abundance in space (interaction term between geographic longitude and latitude, Table 1).

The number of anthropogenic food sources was non-linearly positively correlated with the abundance of black plumage morphs (Table 1; Fig. 3). The cover of tall buildings correlated with the abundance of blue and mixed plumage morphs: blue morph abundance was the highest at moderate values of tall building cover, and mixed plumage morph abundance increased with cover (Table 1; Fig. 3). The distance from the city center was negatively correlated with the blue plumage morphs but non-linearly positively with the abundance of white plumage morphs (Table 1; Fig. 3). The abundance of each morph was correlated with the abundance of the other morphs (Table 1; Fig. 3). Moreover, spatial autocorrelation was observed for the abundance of black, red, white, and mixed plumage morphs (Table 1; Fig. 3).

### Variables correlated with the proportion of plumage color morphs

**Fig. 4:**
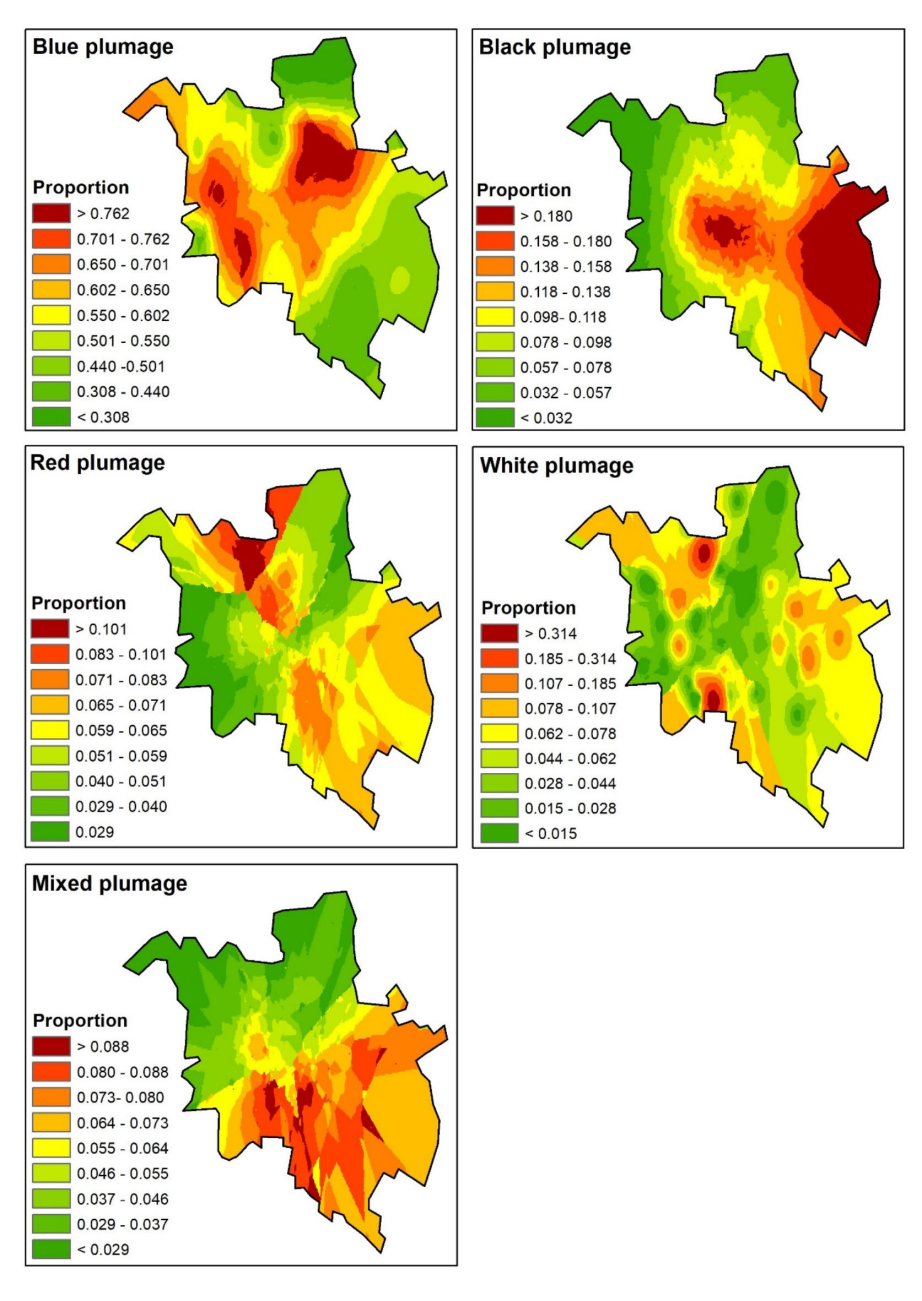
Interpolated proportion of different plumage color morphs of feral pigeon in Poznań. Calculations were done in ArcGIS Desktop: Release 10.8.2 (ESRI 2024). Available from https://www.arcgis.com/.

Contrary to the abundance, the proportion of plumage color morphs was concentrated around the city center only in blue and black morphs (Fig. 4). The proportion of red and white plumage morphs was the highest on the city edges, whereas the proportion of mixed plumage morphs was the highest in the southeastern part of the city (Fig. 4).

**Fig. 5:**
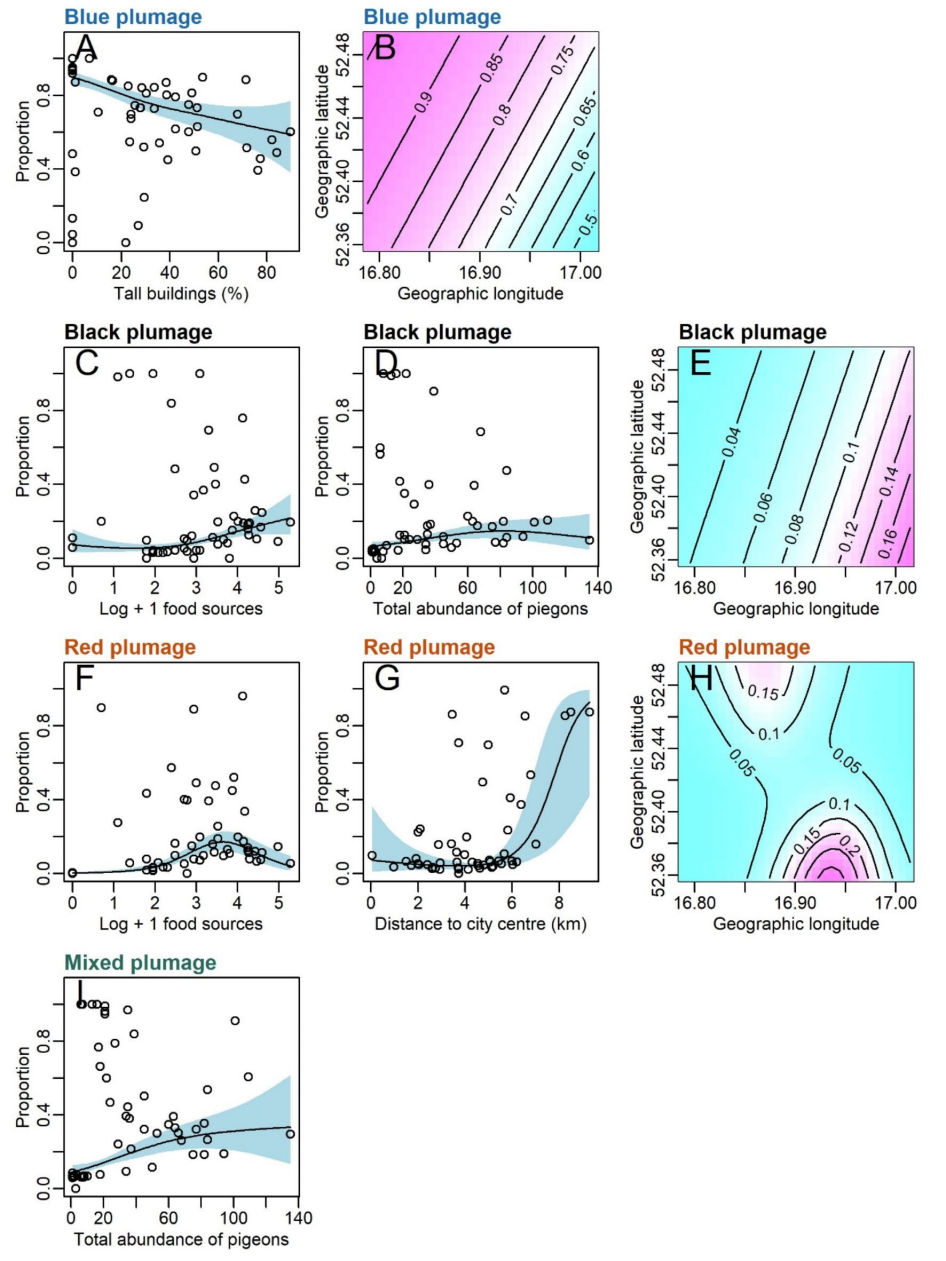
Variables correlated with the proportion of blue plumage morph (**A**,**B**), black plumage morph (**C**–**E**) red plumage morph (**F**–**H**), and mixed plumage morph (**I**). Results from generalized additive models with beta error distribution (Table 2). Isolines in graphs B, E, and H indicate changes in the proportion of the morphs in space (interaction term between geographic longitude and latitude, Table 2).

The GAM indicated that the number of anthropogenic food sources was non-linearly positively correlated with the proportion of black plumage morphs and non-linearly associated with the proportion of red plumage morphs, peaking at moderate values of these sources (Table 2; Fig. 5). The cover of tall buildings was negatively associated with the proportion of blue plumage morphs (Table 2; Fig. 5). The distance to the city center was positively correlated with the proportion of red plumage morphs (Table 2; Fig. 5). The total abundance of all pigeons was positively correlated with the proportion of mixed plumage morphs (Table 2; Fig. 4). Moreover, spatial autocorrelation was observed in the proportions of the blue, black, and red plumage morphs (Table 2; Fig. 5). None variable affected the proportion of white morphs (Table 2; Fig. 5).

### Plumage color morph diversity

**Fig. 6:**
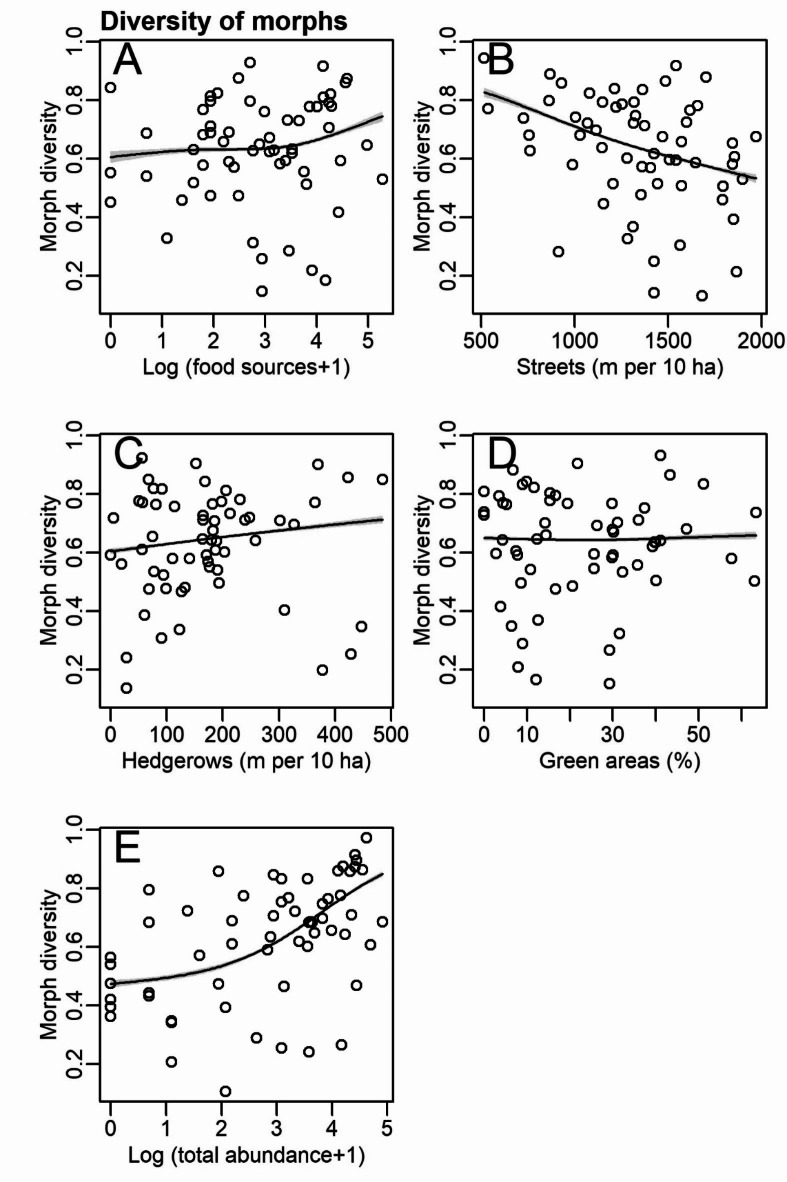
Variables correlated with diversity (Simpson index) of plumage morphs of feral pigeons in Poznań. Results from generalized additive models with beta error distribution (Table 1).

Plumage color diversity (the probability that two randomly chosen pigeons belong to different plumage color morphs) was positively correlated with the number of food sources, density of hedgerows, and total abundance of pigeons but negatively correlated with street density (Table 1; Fig. 6). Moreover, plumage color diversity was slightly and non-linearly correlated with green area cover (Table 1; Fig. 6).

## Discussion

In the present study, we analyzed the numerical response of plumage color polymorphisms in feral pigeons to various variables in an urban environment. The primary finding was that the abundance and proportion of different plumage color morphs in feral pigeons responded differentially to certain environmental variables but responded similarly to social variables (the abundance of other plumage morphs or total abundance). Moreover, we quantified the plumage color diversity index, which was best explained by the variables associated with food sources, green areas, street density, and total pigeon abundance in the urban environment. This indicates that the composition of the urban landscape may determine plumage color diversity and alter the relative abundance of different morphs. Overall, our results validate the behavioral adaptability of feral pigeons, enabling them to effectively exploit urbanized environment^47,55^.

The availability of anthropogenic food sources was positively but non-linearly correlated with the abundance of black morphs, the proportion of red morphs, and the plumage morph diversity index. Food availability notably increases the abundance and reproductive rate of feral pigeons in towns and cities^56^. Black pigeons exhibit different behaviors compared to other plumage color morphs. Black morphs are more aggressive and active^39,40^ and thus have higher energy expenditure, food consumption, and energy demand than other morphs. Moreover, black coloration may sequester more toxic heavy metals to plumage pigments via chelation^27,31,57^. However, this process incurs additional energy expenses^30,32^. The black plumage strengthens feathers, allowing quicker flights. Lofts et al.^58^ demonstrated that the gonads of melanic pigeons remain active throughout winter, unlike those of the blue morph^59^, which may be explained by rapid heating by the black plumage in the urban environment during this period.

The proportion of red morphs was positively correlated with the number of food sources. The behavior of this morph and the potential factors responsible for its occurrence remain largely unknown. Pheomelanin production requires much more energy and trace element availability than eumelanin production^60^. This may explain the relative rarity of red plumage morphs in urban areas.

The positive correlation between the availability of food sources and the abundance or proportion of black and red morphs could further explain the positive correlation between plumage color diversity and food source availability. These morphs are rare; therefore, if their abundance increases in relation to the most common blue morph, the diversity index value increases. The index reaches its maximum value when different plumage color morphs have equal abundances.

The plumage diversity of pigeons was negatively affected by high street density. Pigeons often use road puddles for bathing after rainfall^47^. Pigeons may also congregate near streets to feed, as demonstrated by Rose et al.^45^. Road surfaces can absorb and retain significant amounts of solar heat, resulting in an average temperature 7–10 °C higher than the surrounding area^61^ and may attract pigeons, particularly during the colder months. However, considering the positive association between the total abundance of pigeons and the diversity index, this should actually increase the diversity of the plumage color morphs. City streets can potentially be ecological traps, increasing the likelihood of car collisions and causing notable deaths among various species in urban environment^50,62^. Some studies have revealed that bird mortality increases with traffic^41,42^. If road mortality is mainly associated with less common plumage morphs (black), the relative abundance (proportion) of the most common morph increases, decreasing the diversity index. However, neither the abundance nor the proportion of morphs was statistically associated with street density. Therefore, the negative impact of street density on plumage color diversity may result from behavioral responses and avoidance of areas with high street density. Car traffic increases disturbances and generates a high level of noise pollution, which is avoided by birds^43,44,63–65^.

The morph diversity index was positively correlated with the density of hedgerows and the cover of green areas. These variables describe the availability of semi-natural foraging habitats. Birds may find natural food sources (weed seeds) in such areas. For example, *Ligustrum vulgare* is a predominant species planted in hedgerows^66^, and its fruit is occasionally consumed by feral pigeons (unpublished data). Moreover, many weeds grow in hedgerows^67^ and produce seeds that are frequently eaten by pigeons^68^. Second, people frequently visit these places and feed on birds, particularly in city parks. Remnant semi-natural green areas, such as lawns, trees, and weedy vegetation, are positively correlated with the abundance of many other bird species in urban areas^69,70^.

Among other environmental variables, building size was significantly associated with the abundance of certain plumage color morphs. The abundance and proportion of blue plumage morphs peaked at a moderate level and decreased with tall building cover. Many pigeons nest mainly in taller buildings in residential areas, possibly to avoid predation and disturbance^71,72^. Tall buildings with ledges are common roosting and loafing sites^73^. Harris et al.^74^ found a positive linear association between building height and the abundance of feral pigeons. Tang et al.^72^ found a positive correlation between the number of tall buildings and pigeon density in Singapore. In our study, this association was non-linear, indicating that low and high values of tall building cover decreased the abundance of blue plumage morphs but not of the others. This may partially explain why the proportion of blue morphs decreased with the coverage of tall buildings. Notably, the blue morph resembles its ancestors, wild rock pigeons, which nest on cliffs and rock ledges. Tall buildings are believed to be analogous to cliffs and rock ledges^75^.

Feral pigeons are predominantly located in city centers^76–78^ but are rarely observed on the outskirts of cities^79,80^. However, in our study, formal statistical tests showed that only blue and white morphs showed this dependence after accounting for the effects of other variables. The black morph was expected to show the strongest dependence on the distance to the city center, as the urban environment favors this morph^20,81,82^. The association between distance to the city center and the abundance of consecutive morphs is possibly modified on a larger spatial scale by small towns and villages around Poznań. Pigeons inhabit these locations; therefore, the city of Poznań cannot be considered a habitat island for this species. All plumage color morphs showed spatial autocorrelation except for blue. This indicates that the abundances of these morphs were similar at adjacent sites^83^. Feral pigeons are resident species with strong philopatry in the places where they hatch^84^. This indicates that they exhibit limited movement, which may explain spatial autocorrelation^85,86^. However, the lack of a significant autocorrelation in the blue morph remains challenging to explain. These morphs may be dispersive because they are the most abundant and density-dependent dispersal has been observed in feral pigeons^84^. However, we are unaware of any studies comparing the movements (distance and flight activity) among plumage color morphs in feral pigeons.

One challenge is determining whether the observed differences in response to environmental characteristics, which subsequently result in differences in abundance and distribution, are due to natural selection or simply behavioral responses without further consequences.

Feral pigeons are generally sedentary and cover small distances. However, the distance between the nearest plots (122–2745 m) was within the range of the daily flight distances of some pigeons. Carlen and Munshi-South^87^ found that pigeons within 25 km were highly related. Their analysis detected higher-than-expected gene flow under an isolation-by-distance model within the studied northeastern megacity. Considering the possibility of some birds flying between different areas of the city and the high heritability of plumage color, the observed patterns in abundance and proportion of color morphs likely reflected behavioral plasticity rather than natural selection, favoring different plumage color morphs under specific urban landscape compositions. Highly positive associations among the abundances of different plumage color morphs may also hinder natural selection.

### Study limitations

Our study has certain limitations that should be considered when interpreting the results. First, only one city was included. However, this is one of the largest cities in Poland, with a typical structure (historic old town in the center, surrounded by housing estates, intersected by areas with family houses, and then many semi-natural habitats on the outskirts) of the towns and cities in this part of Europe. Therefore, the results from our study area may be representative of those from other European cities. Second, counts were performed for one year (2010) and during the breeding season (summer). Different factors may affect plumage morph abundance during different years and non-breeding periods, as observed in the plumage color morphs of other species^88,89^. Third, we did not classify the plumage color polymorphisms into more detailed categories^19^. This limited the interpretation and estimation of the plumage diversity. As mentioned previously, conducting a detailed classification was not feasible owing to the involvement of several volunteers, which could have introduced bias. We did not formally test the repeatability of the morph classification; however, it performed consistently with the five plumage morphs. Observers were trained to classify any uncertain observations as a mixed morph. However, mixed plumage morphs constituted only 6.2% of all recorded pigeons. Notably, despite our broad classification, the statistical model for plumage color diversity explained a notably high level of variance. However, a detailed classification of morphs based on color and the distribution of color patches in several cities is required.

## Conclusion

This study provides information on plumage color polymorphisms in feral pigeons in one of the largest Polish cities. Overall, the abundance, proportion, and diversity of plumage color morphs were primarily influenced by a combination of urban infrastructure, available food supplies (human-made and partially natural), and geographical autocorrelation. However, the impact of the urban environment on the different morphs varied. Natural selection unlikely sustained these differential responses because of the relatively small study area. Moreover, possible social attraction among morphs may diminish the niche separation imposed on different morphs through certain features of urban environments.

## Electronic supplementary material

Below is the link to the electronic supplementary material.

Supplementary Material 1

Supplementary Material 2

## Data Availability

Data is provided within supplementary information files:1. “Dane.xlsx” - Excel file with data and description.2. “Pigeons_plumage_polymorphism.R.docx” - R script that contains codes for data handling, statistical analysis and visualisation.
