# Supplementary material for: Environmental and social correlates of the plumage color polymorphism in an urban dweller, feral pigeon (Columba livia f. domestica)
Source: Sci Rep. 2024 Dec 28;14:31400. doi: 10.1038/s41598-024-82937-z (PMC11682311; doi:10.1038/s41598-024-82937-z)
Supplement: Supplementary file 2 — Supplementary Material 2 [file 41598_2024_82937_MOESM2_ESM.docx]

**R script to repeat statistical analyses. Copy all below to R.**

#1. PREPARING DATA ========

# _________________________

# set your own working directory directory

setwd("D:/DATA/Golad_badania/ostatecznaANALIZA")

# load required libraries

library(readxl)

library(forcats)

library(ggplot2)

library(dplyr)

library(tidyr)

library(metan)

library(PerformanceAnalytics)

library(openxlsx)

library(officer)

library(mgcv)

library(TeachingDemos)

library(vegan)

library(flextable)

library(plyr)

library(forcats)

# Read data from supplement

Data <- read_excel("Dane.xlsx", sheet = "analysis_gam")

str(Data)

# Calculating general statistics for each plumage color morhp

# General abundance

Total_abundance <- Data %>%

select("Mean_Blue",

"Mean_White",

"Mean_Black",

"Mean_Red",

"Mean_Mixed")

colnames(Total_abundance)<- c("Blue","White", "Black", "Red", "Mixed")

# Change into long format

Total_abundance1 <- Total_abundance %>%

pivot_longer(cols = c("Blue",

"White",

"Black",

"Red",

"Mixed"), names_to = "Morphs", values_to = "Abundance")%>% as.data.frame()

Total_abundance1$Morphs <- factor(Total_abundance1$Morphs,

levels = c("Mixed", "White","Red", 'Black', "Blue"))

str(Total_abundance1)

# summary statistics

Total_abundance2 <-

Total_abundance1 %>%

group_by(Morphs) %>%

summarize(Mean = mean(log(Abundance+1)),

SD = sd(log(Abundance+1)),

ymin = exp(Mean-SD),

ymax = exp(Mean+SD),

Mean_t = exp(Mean),

MAX = max(Abundance),

MIN = min(Abundance)) %>% as.data.frame()

# select continuous variables

Data_selected <- Data %>%

select("Mean_Blue",

"Mean_White",

"Mean_Black",

"Mean_Red",

"Mean_Mixed",

"Total_pigeons",

"Food_resources",

"Dens_hedgerows",

"Dens_streets",

"Perc_green_areas",

"Distance_city_centre",

"N_schools",

"Perc_tall_buildings")

head(Data_selected)

# preparing data to ggplot - changing to long format

Data_long <- Data_selected %>%

pivot_longer(cols = c("Mean_Blue",

"Mean_White",

"Mean_Black",

"Mean_Red",

"Mean_Mixed",

"Total_pigeons",

"Food_resources",

"Dens_hedgerows",

"Dens_streets",

"Perc_green_areas",

"Distance_city_centre",

"N_schools",

"Perc_tall_buildings"),

names_to = "Variable",

values_to = "Value")

# check distribution of variables

histograms <- ggplot(Data_long, aes(x = Value))+

geom_histogram(fill= "steelblue", alpha = 0.8)+

facet_wrap(.~ Variable, scale = "free", ncol = 3)

histograms

# check correlation among variables

chart.Correlation(Data_selected, histogram=TRUE, pch=16,

method = "spearman")

#2. GAM MODELS FOR ABUNDANCE --------

# calculation number of non-blue morphs

Data$N_non_blue <- Data$Total_pigeons - Data$Mean_Blue

###2.1. GAM model for blue morph -------

m_blue <- gam(Mean_Blue ~

s(log(Food_resources+1), k = 4) +

s(Dens_streets, k =5) +

s(Dens_hedgerows, k = 4)+

s(Perc_green_areas, k = 4)+

s(N_schools, k = 4)+

s(Perc_tall_buildings, k = 4) +

s(Distance_city_centre, k =4)+

s(X,Y, k = 8) +

s(N_non_blue, k = 4),

select = T,

family = nb(),

data = Data)

summary(m_blue)

# plot(m_blue)

gam.check(m_blue)

# saving data to word file

save_as_docx(as_flextable(m_blue),

path = "BLUE.docx",

align = "left")

###2.2. GAM model for black morph -------

# calculation abundance of non-black morphs

Data$N_non_black <- Data$Total_pigeons - Data$Mean_Black

# GAM black

m_black <- gam(Mean_Black ~

s(log(Food_resources+1), k = 4) +

s(Dens_streets, k =4) +

s(Dens_hedgerows, k = 4)+

s(Perc_green_areas, k = 4)+

s(N_schools, k = 4)+

s(Perc_tall_buildings, k = 4) +

s(Distance_city_centre, k =4)+

s(X,Y, k = 8) +

s(N_non_black, k = 4),

select = T,

family = nb(),

data = Data)

summary(m_black)

gam.check(m_black)

plot(m_black)

#save data in Word file

save_as_docx(as_flextable(m_black),

path = "BLACK.docx",

align = "left")

###2.3. GAM model for red morph ----

# calculation number of non-red morphs

Data$N_non_red <- Data$Total_pigeons - Data$Mean_Red

m_red <- gam(Mean_Red ~

s(log(Food_resources+1), k = 4) +

s(Dens_streets, k =4) +

s(Dens_hedgerows, k = 4)+

s(Perc_green_areas, k = 4)+

s(N_schools, k = 4)+

s(Perc_tall_buildings, k = 4) +

s(Distance_city_centre, k =4)+

s(X,Y, k = 10)+

s(N_non_red, k = 4),

select = T,

family = nb(),

data = Data)

summary(m_red)

gam.check(m_red)

plot(m_red)

# saving results in Word file

save_as_docx(as_flextable(m_red),

path = "RED.docx",

align = "left")

###2.4. GAM model for white morph -------

# calculation abundance of non-white morphs

Data$N_non_white <- Data$Total_pigeons - Data$Mean_White

m_white <- gam(Mean_White ~

s(log(Food_resources+1), k = 4) +

s(Dens_streets, k =6) +

s(Dens_hedgerows, k = 4)+

s(Perc_green_areas, k = 4)+

s(N_schools, k = 4)+

s(Perc_tall_buildings, k = 4) +

s(Distance_city_centre, k =4)+

s(X,Y, k = 8)+

s(N_non_white, k = 4),

select = T,

family = nb(),

data = Data)

summary(m_white)

gam.check(m_white)

plot(m_white)

# save results in Word file

save_as_docx(as_flextable(m_white),

path = "WHITE.docx",

align = "left")

###2.5. GAM model for mixed morph -------

# calculation abundance of non-mixed morphs

Data$N_non_mixed <- Data$Total_pigeons - Data$Mean_Mixed

m_mixed <- gam(Mean_Mixed ~

s(log(Food_resources+1), k = 4) +

s(Dens_streets, k =4) +

s(Dens_hedgerows, k = 4)+

s(Perc_green_areas, k = 4)+

s(N_schools, k = 4)+

s(Perc_tall_buildings, k = 4) +

s(Distance_city_centre, k =4)+

s(X,Y, k = 15)+

s(N_non_mixed, k = 4),

select = T,

family = nb(),

data = Data)

summary(m_mixed)

gam.check(m_mixed)

plot(m_mixed)

# save results in Word file

save_as_docx(as_flextable(m_mixed),

path = "MIXED.docx",

align = "left")

#3.PLOTTING SIGNIFICANT EFFECTS (Figure 3 in the manuscript) ==================

tiff(filename = "All_morphs_revised2.tif", res = 400,

units = "cm", width = 15, height = 25)

par(mfrow=c(5,3))

par(mai=c(0.4,0.4,0.2,0.1)) # A

plot(m_blue, select= 6, shade = T, pch = 1,

shade.col = "lightblue",

residuals = T,

ylab = "Mean abundance", xlab = "Tall buildings (%)",

shift = coef(m_blue)[1],

trans = exp, seWithMean = F,

ylim = c(0, 90),

rug = F,

mgp=c(1.5,0.5,0))+

mtext("A", side=3, cex= 1.3, adj = 0.05, line = - 1.5)

title(main = "Blue plumage", adj = 0, col.main = "#1c6bb1")

par(mai=c(0.4,0.3,0.2,0.2))

plot(m_blue, select= 7, shade = T, pch = 1,

shade.col = "lightblue", residuals = T,

ylab = "Mean abundance", xlab = "Distance to city centre (km)",

shift = coef(m_blue)[1], seWithMean = F,

trans = exp,

ylim = c(0,90),

rug = F,

mgp=c(1.5,0.5,0))+

mtext("B", side=3, cex= 1.3, adj = 0.05, line = - 1.5)

title(main = "Blue plumage", adj = 0, col.main = "#1c6bb1")

par(mai=c(0.4,0.3,0.2,0.2))

plot(m_blue, select= 9, shade = T, pch = 1,

shade.col = "lightblue",

residuals = T,

ylab = "Mean abundance", xlab = "Abundance of other morphs",

shift = coef(m_blue)[1], trans = exp,

ylim = c(0,90),

seWithMean = F,

rug = F,

mgp=c(1.5,0.5,0))+

mtext("C", side=3, cex= 1.3, adj = 0.05, line = - 1.5)

title(main = "Blue plumage", adj = 0, col.main = "#1c6bb1")

par(mai=c(0.4,0.4,0.2,0.1)) # D, 2 row

plot(m_black, select = 1, shade = TRUE, pch = 21,

shade.col = "lightblue",

residuals = T,

ylab = "Mean abundance", xlab = "Log+1 food sources",

shift = coef(m_black)[1], seWithMean = F,

trans = exp,

rug = F,

ylim = c(0, 20),

mgp=c(1.5,0.5,0))+

mtext(text = "D", side=3, cex= 1.3, adj = 0.05, line = - 1.5)

title(main = "Black plumage", adj = 0)

par(mai=c(0.4,0.3,0.2,0.2)) # E 2 row

plot(m_black, select= 9, shade = T, pch = 1,

shade.col = "lightblue",

residuals = T,

ylab = "Mean abundance", xlab = "Abundance of other morphs",

shift = coef(m_black)[1],

trans = exp, seWithMean = F,

ylim = c(0, 25),

rug = F,

mgp=c(1.5,0.5,0))+

mtext("E", side=3, cex= 1.3, adj = 0.05, line = - 1.5)

title(main = "Black plumage", adj = 0)

par(mai=c(0.4,0.3,0.2,0.2))

vis.gam(m_black, view = c("X","Y"),

plot.type = "contour", color = "cm", type = "response",

mgp=c(1.5,0.5,0),

main = "",ylab = "Geographic latitude", xlab = "Geographic longitude")

mtext("F", side=3, cex= 1.3, adj = 0.05, line = - 1.5)

title(main = "Black plumage", adj = 0)

par(mai=c(0.4,0.4,0.2,0.1))

plot(m_red, select= 9, shade = T, pch = 1, # G, 3rd row

shade.col = "lightblue",

residuals = T,

ylab = "Mean abundance", xlab = "Abundance of other morphs",

shift = coef(m_red)[1],

trans = exp, seWithMean = F,

ylim = c(0, 20),

rug = F,

mgp=c(1.5,0.5,0))+

mtext("G", side=3, cex= 1.3, adj = 0.05, line = - 1.5)

title(main = "Red plumage", adj = 0, col.main ="#c94b0a")

par(mai=c(0.4,0.3,0.2,0.2))

vis.gam(m_red, view = c("X","Y"),

plot.type = "contour", color = "cm", type = "response",

mgp=c(1.5,0.5,0),

main = "",ylab = "Geographic latitude", xlab = "Geographic longitude")

mtext("H", side=3, cex= 1.3, adj = 0.05, line = - 1.5)

title(main = "Red plumage", adj = 0,col.main ="#c94b0a")

par(mai=c(0.4,0.3,0.2,0.2))

plot(1, type = "n", xlab = "",

ylab = "", xlim = c(0, 5),

ylim = c(0, 5), axes = FALSE)

par(mai=c(0.4,0.4,0.2,0.1))

plot(m_white, select= 7, shade = T, pch = 1,

shade.col = "lightblue",

residuals = T,

ylab = "Mean abundance", xlab = "Distance to city centre (km)",

shift = coef(m_white)[1],

trans = exp, seWithMean = F,

ylim = c(0, 15),

rug = F,

mgp=c(1.5,0.5,0))+

mtext("I", side=3, cex= 1.3, adj = 0.05, line = - 1.5)

shadowtext(x = 2.4, y = 16.5, labels = "White plumage",

xpd =T, r = 0.08, cex = 1.05)

par(mai=c(0.4,0.3,0.2,0.2))

plot(m_white, select= 9, shade = T, pch = 1,

shade.col = "lightblue",

residuals = T,

ylab = "Mean abundance", xlab = "Abundance of other morphs",

shift = coef(m_white)[1],

trans = exp, seWithMean = F,

ylim = c(0, 15),

rug = F,

mgp=c(1.5,0.5,0))+

mtext("J", side=3, cex= 1.3, adj = 0.05, line = - 1.5)

shadowtext(x = 33.5, y = 16.5, labels = "White plumage",

xpd =T, r = 0.08, cex = 1.05)

par(mai=c(0.4,0.3,0.2,0.2))

vis.gam(m_white, view = c("X","Y"),

plot.type = "contour", color = "cm", type = "response",

mgp=c(1.5,0.5,0),

main = "",ylab = "Geographic latitude", xlab = "Geographic longitude")

mtext("K", side=3, cex= 1.3, adj = 0.05, line = - 1.5)

shadowtext(x = 16.846, y = 52.502, labels = "White plumage",

xpd =T, r = 0.08, cex = 1.05)

par(mai=c(0.4,0.4,0.2,0.1))

plot(m_mixed, select= 6, shade = T, pch = 1,

shade.col = "lightblue",

residuals = T,

ylab = "Mean abundance", xlab = "Tall buildings (%)",

shift = coef(m_mixed)[1],

trans = exp, seWithMean = F,

ylim = c(0, 20),

rug = F,

mgp=c(1.5,0.5,0))+

mtext("L", side=3, cex= 1.3, adj = 0.05, line = - 1.5)

title(main = "Mixed plumage", adj = 0, col.main = "#286a5d")

par(mai=c(0.4,0.3,0.2,0.2))

plot(m_mixed, select= 9, shade = T, pch = 1,

shade.col = "lightblue", residuals = T,

ylab = "Mean abundance", xlab = "Abundance of other morphs",

shift = coef(m_mixed)[1], seWithMean = F,

trans = exp,

ylim = c(0,20),

rug = F,

mgp=c(1.5,0.5,0))+

mtext("M", side=3, cex= 1.3, adj = 0.05, line = - 1.5)

title(main = "Mixed plumage", adj = 0,col.main = "#286a5d")

par(mai=c(0.4,0.3,0.2,0.2))

vis.gam(m_mixed, view = c("X","Y"),

plot.type = "contour", color = "cm", type = "response",

mgp=c(1.5,0.5,0),

main = "",ylab = "Geographic latitude", xlab = "Geographic longitude")

mtext("N", side=3, cex= 1.3, adj = 0.05, line = - 1.5)

title(main = "Mixed plumage", adj = 0,col.main = "#286a5d")

dev.off()

layout(1)

#4. MORPH DIVERSITY --------

###4.1. Calculating color morph diversity index -----

Subdata <- Data %>%

select(Plot, contains("Mean"))

# Simpson diversity index

simpson <- ddply(Data,~Plot,function(x) {data.frame(SIMPSON=diversity(x[-1], index="simpson"))})

# Shanno diversity index (just for comparison)

shannon <- ddply(Data,~Plot,function(x) {data.frame(SHANNON=diversity(x[-1], index="shannon"))})

# adding calculated indices to the Data table

Data <- inner_join(x = Data, y = (inner_join(simpson, shannon, by = "Plot")), by = "Plot") %>% as.data.frame

View(Data)

# basic histograms for the indices

hist(Data$SIMPSON)

hist(Data$SHANNON)

plot(Data$SIMPSON,Data$SHANNON)

cor.test(Data$SIMPSON,Data$SHANNON)

###4.2. GAM for morph diversity -------

# Simpson index is chosen because its values are easily interpretable

# GAM for diversity of morphs

morph_div <- gam(SIMPSON ~

s(log(Food_resources+1), k = 4) +

s(Dens_streets, k = 4) +

s(Dens_hedgerows, k = 4)+

s(Perc_green_areas, k = 6)+

s(N_schools, k = 4)+

s(Perc_tall_buildings, k = 4) +

s(Distance_city_centre, k =4)+

s(X,Y, k = 10)+

s(log(Total_pigeons+1), k = 4),

select = T,

family = betar(),

method = "REML",

data = Data)

summary(morph_div)

gam.check(morph_div)

plot(morph_div)

# save results in Word file

save_as_docx(as_flextable(morph_div),

path = "DIVERSITY.docx",

align = "left")

###4.3. Plotting significant effects for morph diversity index (Figure 6 in the manuscript) -------

tiff(filename = "morph_div2.tif", res = 400,

units = "cm", width = 10, height = 15)

par(mfrow=c(3,2), mai=c(0.1,0.1,0.1,0.1))

par(mai=c(0.4,0.5,0.2,0))

plot(morph_div, select= 1, shade = T, pch = 1,

shade.col = "lightblue",

residuals = T,

ylab = "Morph diversity", xlab = "Log (food sources+1)",

shift = coef(morph_div)[1],

trans = plogis, seWithMean = T,

rug = F,

mgp=c(1.5,0.5,0))+

mtext("A", side=3, cex= 1.3, adj = 0.05, line = - 1.5)

title(main = "Diversity of morphs", adj = 0)

par(mai=c(0.4,0.4,0.2,0.1))

plot(morph_div, select= 2, shade = T, pch = 1,

shade.col = "lightblue", residuals = T,

ylab = "Morph diversity", xlab = "Streets (m per 10 ha)",

shift = coef(morph_div)[1], seWithMean = T,

trans = plogis,

rug = F,

mgp=c(1.5,0.5,0))+

mtext("B", side=3, cex= 1.3, adj = 0.05, line = - 1.5)

par(mai=c(0.4,0.5,0.2,0))

plot(morph_div, select= 3, shade = T, pch = 1,

shade.col = "lightblue",

residuals = T,

ylab = "Morph diversity", xlab = "Hedgerows (m per 10 ha)",

shift = coef(morph_div)[1], trans = plogis,

seWithMean = T,

rug = F,

mgp=c(1.5,0.5,0))+

mtext("C", side=3, cex= 1.3, adj = 0.05, line = - 1.5)

par(mai=c(0.4,0.4,0.2,0.1))

plot(morph_div, select= 4, shade = T, pch = 1,

shade.col = "lightblue",

residuals = T,

ylab = "Morph diversity", xlab = "Green areas (%)",

shift = coef(morph_div)[1], trans = plogis,

seWithMean = T,

rug = F,

mgp=c(1.5,0.5,0))+

mtext("D", side=3, cex= 1.3, adj = 0.05, line = - 1.5)

par(mai=c(0.4,0.5,0.2,0))

plot(morph_div, select= 9, shade = T, pch = 1,

shade.col = "lightblue",

residuals = T,

ylab = "Morph diversity", xlab = "Log (total abundance+1)",

shift = coef(morph_div)[1], trans = plogis,

seWithMean = T,

rug = F,

mgp=c(1.5,0.5,0))+

mtext("E", side=3, cex= 1.3, adj = 0.05, line = - 1.5)

dev.off()

layout(1)

#6. ANALYSIS OF RELATIVE ABUNDANCE (PROPORTION) OF MORPHS -----------

###6.1. GAM for the proportion of blue morph ----

Data$prop_blue <- Data$Mean_Blue/Data$Total_pigeons

DataNAremoved <- Data[!is.na(Data$prop_blue),] # optional (`gam` function does it anyway)

m_prop_blue <- gam(prop_blue ~

s(log(Food_resources+1), k = 4) +

s(Dens_streets, k =5) +

s(Dens_hedgerows, k = 4)+

s(Perc_green_areas, k = 4)+

s(N_schools, k = 4)+

s(Perc_tall_buildings, k = 6) +

s(Distance_city_centre, k =4)+

s(X,Y, k = 8) +

s(Total_pigeons, k = 4),

select = T,

family = betar(eps = 0.000001),

data = Data)

summary(m_prop_blue)

plot(m_prop_blue)

gam.check(m_prop_blue)

# saving data to word file

save_as_docx(as_flextable(m_prop_blue),

path = "PROPORTION_BLUE.docx",

align = "left")

###6.2. GAM for the proportion of black morph -----

# calculation proportion of black morphs

Data$prop_black <- Data$Mean_Black/Data$Total_pigeons

m_prop_black <- gam(prop_black ~

s(log(Food_resources+1), k = 4) +

s(Dens_streets, k =5) +

s(Dens_hedgerows, k = 4)+

s(Perc_green_areas, k = 4)+

s(N_schools, k = 4)+

s(Perc_tall_buildings, k = 6) +

s(Distance_city_centre, k =6)+

s(X,Y, k = 8) +

s(Total_pigeons, k = 4),

select = T,

family = betar(eps = 0.000001),

data = Data)

summary(m_prop_black)

gam.check(m_prop_black)

plot(m_prop_black)

# saving data to word file

save_as_docx(as_flextable(m_prop_black),

path = "PROPORTION_BLACK.docx",

align = "left")

###6.3. GAM for the proportion of red morph ------

Data$prop_red <- Data$Mean_Red/Data$Total_pigeons

m_prop_red <- gam(prop_red ~

s(log(Food_resources+1), k = 4) +

s(Dens_streets, k =5) +

s(Dens_hedgerows, k = 4)+

s(Perc_green_areas, k = 4)+

s(N_schools, k = 7)+

s(Perc_tall_buildings, k = 4) +

s(Distance_city_centre, k = 4)+

s(X,Y, k = 8) +

s(Total_pigeons, k = 4),

select = T,

family = betar(eps = 0.000001),

data = Data)

summary(m_prop_red)

gam.check(m_prop_red)

plot(m_prop_red)

# saving data to word file

save_as_docx(as_flextable(m_prop_red),

path = "PROPORTION_RED.docx",

align = "left")

###6.4. GAM for the proportion of white morph ----------

Data$prop_white <- Data$Mean_White/Data$Total_pigeons

m_prop_white <- gam(prop_white ~

s(log(Food_resources+1), k = 4) +

s(Dens_streets, k =5) +

s(Dens_hedgerows, k = 4)+

s(Perc_green_areas, k = 4)+

s(N_schools, k = 4)+

s(Perc_tall_buildings, k = 4) +

s(Distance_city_centre, k = 4)+

s(X,Y, k = 8) +

s(Total_pigeons, k = 4),

select = T,

family = betar(eps = 0.000001),

data = Data)

summary(m_prop_white)

gam.check(m_prop_white)

plot(m_prop_white)

# saving data to word file

save_as_docx(as_flextable(m_prop_white),

path = "PROPORTION_WHITE.docx",

align = "left")

###6.5. GAM for the proportion of mixed morph ----------

# calcultaion of proportion of the mixed morphs

Data$prop_mixed <- Data$Mean_Mixed/Data$Total_pigeons

# GAM for mixed morph.

m_prop_mixed <- gam(prop_mixed ~

s(log(Food_resources+1), k = 4) +

s(Dens_streets, k =5) +

s(Dens_hedgerows, k = 4)+

s(Perc_green_areas, k = 4)+

s(N_schools, k = 4)+

s(Perc_tall_buildings, k = 4) +

s(Distance_city_centre, k = 4)+

s(X,Y, k = 8) +

s(Total_pigeons, k = 4),

select = T,

family = betar(eps = 0.000001),

data = Data)

summary(m_prop_mixed)

gam.check(m_prop_mixed)

plot(m_prop_mixed)

# saving data to word file

save_as_docx(as_flextable(m_prop_mixed),

path = "PROPORTION_MIXED.docx",

align = "left")

# Plotting all significant effects on the proportion of different morphs

#7. PLOTTING SIGNIFICANT EFFECTS (Figure 5 in the manuscript)==================

tiff(filename = "Proportion_all_morphs_rev2.tif", res = 400,

units = "cm", width = 15, height = 20)

par(mfrow=c(4,3))

par(mai=c(0.4,0.4,0.2,0.1)) # A

plot(m_prop_blue, select= 6, shade = T, pch = 1,

shade.col = "lightblue",

residuals = T,

ylab = "Proportion", xlab = "Tall buildings (%)",

shift = coef(m_prop_blue)[1],

seWithMean = F,trans = plogis,

rug = F,

ylim =c(0,1.1),

mgp=c(1.5,0.5,0))+

mtext("A", side=3, cex= 1.3, adj = 0.05, line = - 1.5)

title(main = "Blue plumage", adj = 0, col.main = "#1c6bb1")

par(mai=c(0.4,0.3,0.2,0.2)) #B

vis.gam(m_prop_blue, view = c("X","Y"),

plot.type = "contour", color = "cm", type = "response",

mgp=c(1.5,0.5,0),

main = "",ylab = "Geographic latitude", xlab = "Geographic longitude")

mtext("B", side=3, cex= 1.3, adj = 0.05, line = - 1.5)

title(main = "Blue plumage", adj = 0, col.main = "#1c6bb1")

par(mai=c(0.4,0.3,0.2,0.2))

plot(1, type = "n", xlab = "",

ylab = "", xlim = c(0, 5),

ylim = c(0, 5), axes = FALSE)

par(mai=c(0.4,0.4,0.2,0.1))

plot(m_prop_black, select= 1, shade = T, pch = 1,

shade.col = "lightblue",

residuals = T,

ylab = "Proportion", xlab = "Log + 1 food sources",

shift = coef(m_prop_black)[1], trans = plogis,

ylim = c(0,1.1),

seWithMean = F,

rug = F,

mgp=c(1.5,0.5,0))+

mtext("C", side=3, cex= 1.3, adj = 0.05, line = - 1.5)

title(main = "Black plumage", adj = 0)

par(mai=c(0.4,0.3,0.2,0.2))

plot(m_prop_black, select = 9, shade = TRUE, pch = 21,

shade.col = "lightblue",

residuals = T,

ylab = "Proportion", xlab = "Total abundance of piegons",

shift = coef(m_prop_black)[1],

seWithMean = F,

trans = plogis,

rug = F,

ylim = c(0, 1.1),

mgp=c(1.5,0.5,0))+

mtext(text = "D", side=3, cex= 1.3, adj = 0.05, line = - 1.5)

title(main = "Black plumage", adj = 0)

par(mai=c(0.4,0.3,0.2,0.2)) # E 2 row

vis.gam(m_prop_black, view = c("X","Y"),

plot.type = "contour", color = "cm", type = "response",

mgp=c(1.5,0.5,0),

main = "",ylab = "Geographic latitude", xlab = "Geographic longitude")

mtext("E", side=3, cex= 1.3, adj = 0.05, line = - 1.5)

title(main = "Black plumage", adj = 0)

par(mai=c(0.4,0.4,0.2,0.1))

plot(m_prop_red, select = 1, shade = TRUE, pch = 21,

shade.col = "lightblue",

residuals = T,

ylab = "Proportion", xlab = "Log + 1 food sources",

shift = coef(m_prop_red)[1],

seWithMean = F,

trans = plogis,

rug = F,

ylim = c(0, 1.1),

mgp=c(1.5,0.5,0))+

mtext(text = "F", side=3, cex= 1.3, adj = 0.05, line = - 1.5)

title(main = "Red plumage", adj = 0,col.main ="#c94b0a")

par(mai=c(0.4,0.3,0.2,0.2))

plot(m_prop_red, select= 7, shade = T, pch = 1, # G, 3rd row

shade.col = "lightblue",

residuals = T,

ylab = "Proportion", xlab = "Distance to city centre (km)",

shift = coef(m_prop_red)[1],

trans = plogis, seWithMean = F,

ylim = c(0, 1.1),

rug = F,

mgp=c(1.5,0.5,0))+

mtext("G", side=3, cex= 1.3, adj = 0.05, line = - 1.5)

title(main = "Red plumage", adj = 0,col.main ="#c94b0a")

par(mai=c(0.4,0.3,0.2,0.2))

vis.gam(m_prop_red, view = c("X","Y"),

plot.type = "contour", color = "cm", type = "response",

mgp=c(1.5,0.5,0),

main = "",ylab = "Geographic latitude", xlab = "Geographic longitude")

mtext("H", side=3, cex= 1.3, adj = 0.05, line = - 1.5)

title(main = "Red plumage", adj = 0,col.main ="#c94b0a")

par(mai=c(0.4,0.4,0.2,0.1))

plot(m_prop_mixed, select= 9, shade = T, pch = 1,

shade.col = "lightblue",

residuals = T,

ylab = "Proportion", xlab = "Total abundance of pigeons",

shift = coef(m_prop_white)[1],

trans = plogis, seWithMean = F,

ylim = c(0, 1.1),

rug = F,

mgp=c(1.5,0.5,0))+

mtext("I", side=3, cex= 1.3, adj = 0.05, line = - 1.5)

title(main = "Mixed plumage", adj = 0, col.main = "#286a5d")

dev.off()

layout(1)

# ============== THE END ==================
